# Cognitive, Behavioral and Socioemotional Development in a Cohort of Preterm Infants at School Age: A Cross-Sectional Study

**DOI:** 10.3390/pediatric14010017

**Published:** 2022-03-04

**Authors:** Chiara Ionio, Gianluca Lista, Pierangelo Veggiotti, Caterina Colombo, Giulia Ciuffo, Irene Daniele, Marta Landoni, Barbara Scelsa, Enrico Alfei, Stefania Bova

**Affiliations:** 1 CRIdee, Unità di Ricerca sul Trauma, Dipartimento di Psicologia, Università Cattolica, 20123 Milano, Italy; giulia.ciuffo@unicatt.it (G.C.); marta.landoni@unicatt.it (M.L.); 2 Neonatologia, Patologia e Terapia Intensiva Neonatale, Ospedale dei Bambini “Vittore Buzzi”, ASST Fatebenefratelli Sacco, 20154 Milano, Italy; gianluca.lista@asst-fbf-sacco.it (G.L.); caterina.colombo@asst-fbf-sacco.it (C.C.); irene.daniele@asst-fbf-sacco.it (I.D.); 3 Neurologia Pediatrica, Ospedale dei Bambini “Vittore Buzzi”, ASST Fatebenefratelli Sacco, 20154 Milano, Italy; pierangelo.veggiotti@asst-fbf-sacco.it (P.V.); barbara.scelsa@asst-fbf-sacco.it (B.S.); enrico.alfei@asst-fbf-sacco.it (E.A.); stefania.bova@asst-fbf-sacco.it (S.B.)

**Keywords:** preterm birth, school-age, executive function, IQ, social competence

## Abstract

More than 50% of children who survive prematurity have an atypical course of development at school age, as environmental demands become more demanding. This study examines the effects of preterm birth on the cognitive, behavioral and socioemotional development of 185 children at ages five and seven years. Weaknesses were found in attention, working memory, processing speed and the ability to correctly interpret emotions at both ages five and seven. Significant correlations were found in regression and moderation models. These findings suggest that school-age children who were preterm infants are at increased risk of exhibiting impairments in several developmental domains that may affect their overall quality of life.

## 1. Introduction

Modern neonatology is faced with an ever-increasing percentage of preterm infants worldwide and, in particular, higher rates of very low birth weight (VLBW) or extremely low birth weight (ELBW) infants surviving preterm birth. In the 1960s, the World Health Organization referred to preterm infants as newborns born before 37 weeks of gestation or before 259 days have passed since the last menstrual cycle [1]. Studies have shown that preterm birth can be a significant risk factor for developmental impairment [2]. A preterm infant begins his or her extrauterine life in the Neonatal Intensive Care Unit (NICU), where he or she is hypostimulated due to the lack of uterine control and rhythmic stimulation, but also hyperstimulated by the light, the noise of the unit and the medical interventions, which can sometimes be particularly invasive and painful [3]. This situation is stressful for the child, also because he has no or limited physical contact with his parents [3], and for his parents, who may not be physically, emotionally and psychologically ready to welcome the birth. In addition, because of the long hospital stays in the NICU, preterm infants may also be exposed to risks of long-term psychological effects that may manifest later. In fact, longitudinal studies have provided evidence that preterm infants experience more long-term neurodevelopmental problems compared to term infants. Therefore, it is of great importance for researchers to study the impact of these potentially atypical developmental trajectories and the quality of life of these children [4].

Regarding cognitive development, research clearly shows that between 30% and 60% of very preterm infants experience various cognitive challenges and learning difficulties throughout their development [5]. Impairments in the child’s IQ, executive functions and general well-being appear to progress into adulthood [6]. Deficits in executive functions such as attention, inhibition and processing speed have been frequently observed in this population, especially during school age [7]. It is during this time, when the demands of the environment are becoming more pressing and difficult to manage, that these deficits begin to manifest themselves more clearly and prominently [8].

However, to our knowledge, the literature focuses less on the behavioral and socio-emotional development of this population, although there is some evidence of the so-called behavioral phenotype of preterm infants, which is characterized by a high risk for the development of symptoms and disorders related to social problems [9], in combination with internalizing and externalizing problems [10]. These problems appear to be long-term and have a significant impact on the quality of life of this population [11]. In this context, Moreira, Magalhaes and Alves point out that the percentage of children showing behavioral problems at school age is even higher than at pre-school age [12]. This can be explained by the fact that most of the instruments used to study these difficulties are based on the assessments of the parents, whose perceptions become more accurate as the children grow older. At school age, parents seem to be increasingly concerned about their children’s possible deficits and are more aware of developmental expectations for this age group, so they more often compare their children’s development with that of their peers [13]. Several authors suggest that social perception may play an important role in determining social–emotional problems in preterm infants [14]. This skill involves the ability to selectively infer and extract the most important non-verbal social cues in the current situation. Wocadlo and Rieger found that children born very prematurely have problems recognizing facial expressions and that these difficulties seem to be related to their poor social skills [15]. Consistent with these findings, the results of a recent paper by Williamson and Jakobson show that this population has difficulty correctly interpreting other people’s emotions because of an inability to decode non-verbal cues (body movements, facial expressions and situational cues) that could be used for this type of inferencing during social interactions [16]. These problems, which play an important role in these children’s social difficulties, also contribute to the exacerbation of emotional and behavioral problems [17]. Indeed, their difficulty in recognizing relevant social information may lead them to misinterpret different social situations and, consequently, respond with inappropriate behavior [18].

Following the literature and emphasizing the potential risk of long-term maladjustment in this population, the present study examined school-age children’s vulnerability to cognitive, behavioral and socioemotional deficits following preterm birth. Specifically, in the cognitive domain, we hypothesized that preterm infants might have cognitive impairments in IQ and executive functions that affect their academic performance and learning abilities. Following previous literature [19], we hypothesized that cognitive functioning may be poorly explained and predicted by atypical developmental trajectories and physical factors [20]. In addition, we hypothesized that age may have an impact on behavior and that there is a relationship between behavior and deficits in social cognition.

## 2. Materials and Methods

### 2.1. Data Collection

This study is part of a broader longitudinal research project conducted in 2013 in collaboration with CRIdee (Department of Psychology, Catholic University of the Sacred Heart, Milan, Italy) and Buzzi Children’s Hospital in Milan (Italy), following preterm infants from birth to 7 years of age. It was approved by the Ethics Committee of Azienda Ospedaliera, Istituti Clinici di Perfezionamento, Milano (protocol number: 1171, 11 December 2012). The aim of this broad study was to investigate, from a longitudinal perspective, the impact of preterm birth on child development, paying particular attention to the parental couple and, consequently, to the effects and consequences that affect the whole triad. For this reason, families were followed in two phases from the birth of the child until the age of 7 years. The first phase began at the birth of the child, followed by a further assessment at 3 months of age and ended at the completion of the first year of life [21]. The second time point began at age 4 and continued through annual observations until the premature child was 7 years old.

In the present paper, the second phase of the longitudinal study was analyzed, which included a neuropsychological assessment of the children at 4–7 years of age. Before the tests were administered to the children, the parents signed an informed consent form and a privacy statement.

### 2.2. Participants

Participants were 185 preterm infants aged 5 and 7 years (86 males, 99 females; gestational age (GA) = 28.46 ± 2.08; birth weight (BW) = 1044.89 ± 267.37). Exclusion criteria were the presence of congenital anomalies, severe sensory impairments, severe brain injuries and other neurological complications and parents’ lack of Italian language skills. In addition, 42.1% of the babies were twins; 62.8% were delivered spontaneous vaginally, 14.2% vacuum-assisted vaginally and 23% were delivered by caesarean section. In the first minute after birth, the mean Apgar score was 5.68 (SD = 1.620) and in the fifth minute it was 7.78 (SD = 1.102). The number of days spent in the neonatal intensive care unit ranged from 29 to 144 (M = 70.39; SD = 29.44). Given the importance of parents’ role in infant development, we also considered their age and degree. The mean age of the mothers was 36.52 years (SD = 6.165), and that of the fathers was 41.35 years (SD = 6.509). Most mothers had a high school degree (32%) or a bachelor’s degree (32%), 24% had a postgraduate degree and 12% attended high school without having a degree. As for the fathers, most of them had a bachelor’s degree (47.4%), 15.8% had attended secondary school, 15.8% had a high school diploma, 10.5% had a junior high school diploma and finally, the remaining 10.6% had a primary school diploma (5.3%) or attended university (5.3%). These data suggest that they all had a reasonable level of education. Moreover, only 3.8% of the mothers were unemployed, while all the fathers were employed.

### 2.3. Measures

#### 2.3.1. Cognitive Profile

**Wechsler Intelligence Scale for Children-IV (WISC-IV; Italian Edition Curated by Orsini and Pezzuti, 2012)**

The Wechsler Intelligence Scale for Children-IV (WISC-IV) is a clinical instrument designed to assess the cognitive abilities of children between the ages of six and sixteen. The test includes fifteen subtests, ten of which are core tests and five of which are additional optional subtests. The instrument measures the child’s global IQ and thus describes his or her general cognitive ability. In addition to the IQ, the assessment of the child’s profile is complemented and enriched by four indexes: Verbal Comprehension Index, Perceptual Reasoning Index, Working Memory Index and Processing Speed Index. In order to obtain a comprehensive profile of the cognitive development of our seven-year-old children, we decided to administer all the subtests. The scores ranged from 40 to 160. The Cronbach’s alpha for this study was 0.852.

**Wechsler Preschool and Primary Scale of Intelligence, Third Edition (WPPSI-III, Wechsler, 2008)**

The Wechsler Preschool and Primary Scale of Intelligence, Third Edition (WPPSI-III) is a clinical instrument that focuses on the cognitive abilities of children between the ages of two years, six months and seven years, three months. It consists of 14 subtests divided into three groups: the main tests, the additional tests and the optional ones. We administered all the core subtests to our five-year-old children. The results were added to the Verbal Intelligence Quotient (VIQ), the Performance Intelligence Quotient (PIQ), the Processing Speed Quotient (PSQ) and the Full-Scale IQ (FSIQ). The Cronbach’s alpha for these results was 0.76.

**Neuropsychological Evaluation Battery for Developmental Age (BVN 5–11, Tressoldi et al., 2005)**

The BVN is a test battery that examines the main cognitive functions (language, visual perception, memory, practices, attention, higher executive functions, reading, writing and arithmetic) in children aged five to eleven. It analyses the development of cognitive functions under normal conditions and identifies certain developmental and/or acquired pathologies. We chose the Tower of London subtest (TOL), which assesses the ability to design and plan the necessary strategies to solve a problem correctly. It was administered to 7-year-old children. The Cronbach’s alpha for this test battery was 0.454.

**NEPSY-II (Italian Edition Cured by Urgesi, Campanella and Fabbro, 2011)**

The NEPSY-II is a neuropsychological battery of cognitive skills tests for preschoolers and school-aged children between three and sixteen years old. The Italian version consists of 33 subtests related to six different cognitive domains. We decided to use the subtests from the Executive Function & Attention domain, which consists of 3 subtests: Auditory Attention and Response Set (A3), Inhibition (A4) and Grouping Animals (A6). They were administered to seven-year-old children. The Cronbach’s alpha for this test battery was 0.856.

#### 2.3.2. Behavioral and Socio-Emotional Profile

**NEPSY-II (Italian Edition Cured by Urgesi, Campanella and Fabbro, 2011)**

We administered the Social Perception subtests of the NEPSY-II to the seven-year-old children to assess their ability to recognize facial expressions and decode the intentions of others: Theory of Mind (SO1) and Emotion Recognition (SO2). Theory of Mind (SO1) assesses constructs such as beliefs, intentions, deceptions, emotions, fantasy and imagination and the ability to understand that others have thoughts, ideas and feelings differently from one’s own. Emotion Recognition (SO2) assesses the ability to recognize emotional expressions in photographs of children’s faces.

**Child Behavior Checklist (CBCL; Achenbach, 1991; Achenbach and Rescorla, 2000)**

Parents were asked to complete the Child Behavior Checklist for Ages 1.5–5 (CBCL 1.5–5; Achenbach & Rescorla, 2000) and the Child Behavior Checklist for Ages 4–18 (CBCL 4–18; Achenbach, 1991). These questionnaires provide a multi-axial assessment of subjects’ social, emotional and behavioral skills. We used them primarily to assess internalizing and externalizing problems in five- and seven-year-old children. Scores were divided into three categories: normal range (<93rd percentile), subclinical or borderline range (93rd to 97th percentile) and clinical or elevated range (>97th percentile). The Cronbach’s alpha for this assessment was 0.85.

### 2.4. Statistical Analysis

For the cognitive profile, we first compared the mean scores with the clinical cut-off to assess the children’s executive functions and IQ. We also examined correlations between cognitive outcomes and some physical indicators such as birth weight, Apgar scores in the first and fifth minute and hospitalization days. To better investigate the influence of hospitalization days on cognitive performance, we decided to perform a linear regression for the subscales of the WPPSI-III and WISC-IV. To avoid collinearity problems, linear regressions were conducted separately for WPPSI-III and WISC-IV values.

For the behavioral profile, paired-sample *t*-tests were performed to analyze significant differences in behavior when the variable age was included. Finally, following previous studies, we decided to investigate whether there was a moderation effect of behaviors on cognitive outcomes. We ran a moderation model using JAMOVI in which we included internalizing and externalizing symptoms, social problems and the total measured with CBCL after five years as a moderator, hospitalization days as a predictor and IQ overall, assessed with the WPPSI-III, as a dependent variable.

## 3. Results

### 3.1. Cognitive Profile

As for the mean, the results of the scales of WPPSI-III, listed in Table 1, show that the values of all indices are in the average range, except for the quotient of processing speed (M = 94.54), which is in the medium to low range. The results of the scales of the WISC-IV show how the index of working memory (M = 93.94) and the index of processing speed (M = 88.38) differ from the others, positioning themselves in the medium to low range. Finally, we looked at the children’s performance on the Executive Function & Attention subtest of the NEPSY-II. The results were below average for Inhibition (M = 7.9), Auditory Attention (M = 4.1) and Response Set (M = 4.8).

Correlation analysis showed a positive correlation between weight and total intellectual quotient (TQ) of the WPPSI-III (five-year-old children; r = 0.221, *p* < 0.05) and between weight and the WPPSI-III QPV (r = 0.287, *p* < 0.01) (Table 2. Furthermore, significant correlations were found between hospitalization days and total intelligence quotient (TQ) (r = 0.945, *p* < 0.05), verbal intelligence quotient (VIQ) (r = 0.881, *p* < 0.05) and performance intelligence quotient (PIQ) (r = 0.925, *p* < 0.05). No significant correlations were found between the Apgar scores and the WPPSII-III scales (*p* > 0.05). 

Finally, significant regression equations were found. The first regression results (F (1, 3) = 17.9, *p* = 0.024) showed that hospitalization days explained 85% of the variance for the performance quotient (TQ) of the WPPSI-III scale (R^2^ = 0.85, F (2, 55) = 5.56, *p* < 0.01). Hospitalization days significantly predicted the performance quotient IQ after five years (β = 0.387, *p* < 0.05). Similarly, we found that hospitalization days predicted the Verbal Intelligence Quotient (VIQ) (β = 0.223, *p* < 0.05) with a model (F = (1, 3) = 10.4, *p* = 0.048) which explained 77% of the variance (R^2^ = 0.77). Hospitalization days also predicted IQ overall (β = 0.326, *p* < 0.05; F (1, 3) = 25.1, *p* = 0.015), explaining 89% of the variance (R^2^ = 0.89) (Table 3). No significant regression models were found between the WPPSI-III scales and child indicators (*p* > 0.05).

### 3.2. Behavioral and Socio-Emotional Profiles

To assess the behavior of the children, we first considered their scores on the CBCL 1.5–5 and CBCL 4–18 questionnaires, which were in the average range. The results are presented in Table 4. The difference between the mean scores for internalizing and externalizing was not significant (*p* < 0.05), while a significant difference occurred for the Total Problems scale. In addition, significant correlations were found between IQ and higher scores for social problems (r = −0.445, *p* < 0.01), IQ and higher scores for ToM (r = 0.764, *p* < 0.001) and ToM and executive function (r = 0.387, *p* < 0.05) (Table 3). 

**Figure 1:**
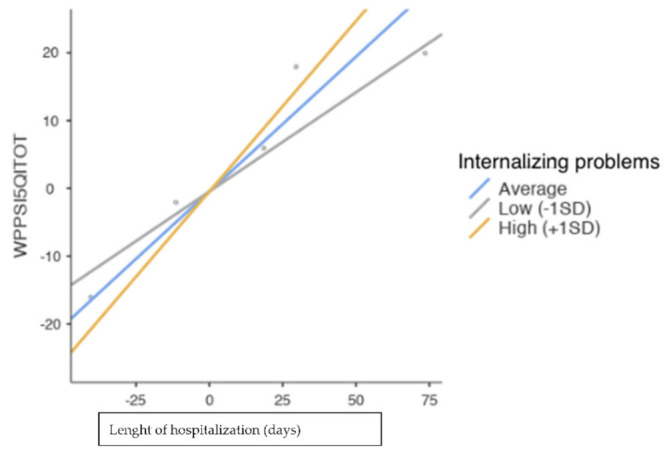
Simple Slope Plot.

No significance was found in moderation models for externalizing symptoms, social problems and total CBCL score (*p* > 0.005). Significant predictive support was found for both hospitalization days (*p* < 0.01) and internalizing symptoms (*p* < 0.01) on the CBCL. The interaction between the two variables was also significant (*p* < 0.01). Both low (b = 0.292, s.e. = 0.058, *p* < 0.01) and high (b = 0.503, s.e. = 0.060, *p* < 0.01) levels of internalizing symptoms were significant in predicting IQ overall. As Figure 1 shows, children with mild internalizing symptoms had a better total IQ score with low hospitalization days, which increased over the course of hospitalization days. Children with severe internalizing symptoms had a lower IQ score at baseline that increased over time.

## 4. Discussion

The aim of this study was to examine children’s cognitive, behavioral and socioemotional development. In particular, regarding the cognitive profile, the analysis showed that the scores obtained by the five-year-olds on the WPPSI-III were all in the average range (90–109). However, the mean value of the processing speed quotient (M = 94.54) was slightly different from the others and was in the middle to low range. These results suggest that preterm infants may develop learning difficulties and have difficulty in performing cognitive and perceptual tasks.

Based the correlational analyses performed, it appears that the children’s IQ increased with increasing birth weight and hospitalization days, and that their processing speed seems to be better with increasing birth weight. These initial results seem to be consistent with what has been reported in the international literature, in which it is highlighted that this population tends to have deficits in certain cognitive domains from preschool age and that this is generally associated with low birth weight. Contrary to expectations, no significant correlations were found between index scores and gestational age [2,20]. In addition, scores of seven-year-olds on the WISC-IV appeared to be consistent with those of five-year-olds on the WPSSI-III. Although all scores were in the average range (85–115), the indices for working memory and processing speed appeared to be more deficient, confirming the possibility that these children may have difficulty learning, retaining or processing information at school age. As for the IQ scores, the average scores at ages five and seven might be related to the schooling of the parents [19]. As we know, the parents of the children in our sample had a relatively high level of education, as most of them had a college degree. According to Mento and Bisiacchi [20], the hypothesis could be that their level of education provides their children with a developmentally stimulating environment that can counteract the effects of biological deficits. Finally, days of hospitalization have been shown to predict cognitive outcomes. It is well known that a prolonged stay of a preterm infant in the stressful environment of the NICU increases the risk of lifelong disability [22]. Thus, a growing number of studies associate repeated neonatal stress with poor neurological outcomes [23].

However, in our study, regression models showed a positive relationship between hospitalization days and cognitive outcomes, i.e., the longer the hospitalization, the better the outcomes. Similarly, recent evidence suggests that developmental care in the NICU significantly affects the mental development of preterm infants by improving their cognitive development index (MDI) [24]. In Kleberg’s work [25], the MDI of infants receiving care according to the Newborn Individualized Developmental Care and Assessment Program (NIDCAP) was higher than the corresponding score in control children, suggesting that NIDCAP-based care may have a positive impact on the cognitive development of very preterm infants. One of the aims of this program is to enhance parents’ abilities to identify and respond to their infants’ cues. By becoming more sensitive to the baby’s needs, they are thus able to provide adequate stimulation [24].

It could be hypothesized that this improved ability may strengthen parent–infant interaction even after discharge from hospital and enhance joint parent–infant attention activities considered beneficial to long-term cognitive development [26]. Although the preterm infants in this study did not receive a structured NIDCAP program, it is conceivable that some of the individual interventions they underwent (e.g., Kangaroo mother care) may explain our results. However, further research is needed to better explain these findings.

Regarding the behavioral profile, our results showed that, contrary to our expectations, the scores of the preterm infants were in the average range at both five and seven years of age. However, it should be noted that our data were based on indirect measurements using questionnaires completed by parents (CBCL 1.5–5 and CBCL 4–18). For this reason, they may be less reliable and further investigation may be required. For this purpose, the SOLE VLBWI questionnaire, a recently validated self-report instrument developed to assess the quality of life of school-age children born preterm with a VLBWI, can be used [27].

A significant difference was discovered in the overall scale of perceived problems for the age variable. Parents appeared to perceive more problems in their children at age seven than at age five. In fact, parents’ perceptions appeared to become more accurate as children aged [12]. Parents are more concerned about potential deficits and become more aware of developmental expectations during the school years [13]. In addition, our results also revealed a moderating effect that internal behavioral symptoms have in altering the relationship between hospital stay length and IQ. Indeed, poor cognitive performance in preterm infants has been found to be strongly associated with behavioral problems, confirming previous findings. Few studies have examined whether behavior can explain differences in the frequency of cognitive problems between preterm and term infants [28]. In a cohort of 194 preterm infants studied at age five years, lower IQ was associated with more hyperactive behavior and inadequate social skills [29]. Literature shows that hospitalization from birth represents a significant risk factor in children born preterm [30,31], as in the general population (e.g., childhood illness and chronic physical conditions) [32]. Our findings seem to move in this direction, however, more detailed research and studies comparing these results with those of a control sample are needed to explain this moderation effect.

With regard to the socioemotional profile, we have paid particular attention to the area of social cognition, in which this population seems to be more vulnerable. Several authors suggest that social cognition may play a crucial role in determining socio-emotional problems in preterm infants [14]. Our analysis revealed that the scores obtained from the Theory of Mind subtest (NEPSY-II) were in the average range (8–12), while performance in emotion recognition was below average. This result confirms previous findings showing that preterm infants may have greater difficulty interpreting the emotions of others [16]. This misinterpretation could also exacerbate emotional and behavioral problems [2]. Finally, we examined the relationship between cognitive impairment and socioemotional outcomes. We found a positive correlation between IQ and Theory of Mind; high IQ scores were associated with higher ToM scores. We also found a negative correlation between IQ and social problems (CBCL 4–18). Regarding executive functions, we found a positive correlation between TOL (BVN 5–11) and Theory of Mind; higher TOL subtest scores were associated with higher ToM scores. There was also a positive correlation between the Inhibition subtest (NEPSY-II) and Theory of Mind; higher scores on the inhibition subtest were associated with higher scores on ToM. Our findings support the hypothesis that adaptive social and emotional functioning is closely related to the functioning of higher-level cognitive processes, such as executive functions, which are used to decode social and emotional information and develop knowledge about the social world [33]. As we have said, these difficulties may have a cascading effect. A child who does not properly understand the social context, may engage in inappropriate behavior [34]. Further research should be conducted to clarify the causal relationship between these variables.

## 5. Strengths and Limitations

This study has some limitations. First, as mentioned earlier, the behavioral profile data were collected by indirect measures using questionnaires completed by parents (CBCL 1.5–5 and CBCL 4–18). For this reason, they may be less reliable and further research is needed. Second, there was a lack of a control group of term infants due to a high dropout rate during follow-up. Third, we did not evaluate emotional and cognitive capabilities of parents that may have played an important protective or risk role in children’s capabilities/difficulties.

Overall, this study highlights the importance of being alert to potential deficits associated with preterm birth over the long-term and the importance of assessing the cognitive and behavioral domains of preterm infants. 

These findings provide an opportunity to implement interventions to improve outcomes for these infants during the period of maximal plasticity. These findings could also be a starting point for future research. They underscore the importance of structuring follow-up interventions for these infants to address the socioemotional and behavioral domains that are often examined.

## 6. Conclusions

Early identification of behavioral and cognitive problems in very preterm children should be encouraged to address them appropriately and promptly, especially since very preterm or very low birth weight infants are also at increased risk for attention-deficit/hyperactivity disorder, depression and anxiety in adolescence. 

Weaknesses were found in attention, working memory, processing speed and the ability to correctly interpret emotions at both ages five and seven. These findings support early screening for these complications in extremely preterm infants, including those who are less immature and do not have cognitive impairments.

## Figures and Tables

**Table 1:** Means and standard deviations.

Variables	Mean	SD	Range
**WPPSI-III Indices**			
FSIQ	104.68	14.55	64–128
VIQ	108.68	14.73	38–133
PIQ	100.29	14.52	29–138
PSQ	94.54	16.22	14–132
**WISC-IV Indices**			
FSIQ	101.72	14.78	64–128
VCI	99.66	20.96	41–140
PRI	99.22	21.93	34–124
WMI	93.94	23.05	19–121
PSI	88.38	23.80	11–129
**BVN5-TOL**	97.85	22.52	11.5–118.2
**CBCL 1.5–5**			
Total Problems	1.65	2.93	1–3
Internalizing Problems	1.32	0.66	1–3
Externalizing Problems	1.43	1.51	1–3
**CBCL 4–18 Scores**			
Total Problems	1.15	0.51	1–3
Internalizing Problems	1.45	0.75	1–3
Externalizing Problems	1.15	0.51	1–3
Social problems	1.10	0.40	1–3

Note: Mean and standard deviation obtained from five- and seven-year-old children. WPPSI-III: Wechsler Preschool and Primary Scale of Intelligence, Third Edition: Verbal Intelligence Quotient (VIQ), Performance Intelligence Quotient (PIQ), Processing Speed Quotient (PSQ) and Full-Scale IQ (FSIQ). Wechsler Intelligence Scale for Children-IV (WISC-IV): Verbal Comprehension Index, Perceptual Reasoning Index, Working Memory Index, Processing Speed Index. Neuropsychological Evaluation Battery for Developmental Age (BVN 5–11). Child Behavior Checklist (CBCL).

**Table 2:** Correlation analysis.

	1	2	3	4	5	6	7	8	9	10	11	12	13	14	15	16
1. WPPSI-III QI TOT	— —															
2. WPPSI-III VIQ	0.780 **	— —														
3. WPPSI-III PIQ	0.765 **	0.273 *	— —													
4. WPPSI-III QVP	0.459 **	0.098	0.380 **	— —												
5. Hospital days	0.945 *	0.881 *	0.925 *	0.597	— —											
6. Weight	0.221 *	0.184	0.079	0.287 **	_0.523 **	— —										
7. Apgar 1	0.055	0.197	0.038	0.115	_0.627 **	−0.1	— —									
8. Apgar 5	0.109	0.194	0.029	0.090	−0.318	−0.1	1.000 **	— —								
9. WISC-IV QI	0.485 **	0.249	0.388 *	0.353	NaN	0.038	0.142	−0.062	— —							
10. WISC-IV ICV	0.384 *	0.506 **	0.063	0.408 *	NaN	0.048	−0.011	0.042	0.311	— —						
11. WISC-IV IRP	0.326	0.186	0.248	0.485 **	NaN	0.153	0.054	0.077	0.285	0.724 **	— —					
12. WISC-IV IML QI Total	0.025	0.133	0.184	0.304	NaN	0.104	0.020	0.038	0.103	0.785 **	0.688 **	— —				
13. WISC-IV IVE	0.258	0.071	0.212	0.333	NaN	0.109	0.173	0.016	0.245	0.583 **	0.680 **	0.717 **	— —			
14. CBCL Social QI Total	0.261	0.160	0.155	0.227	NaN	−0.14	−0.115	0.097	−0.445 **	−0.006	−0.006	−0.008	−0.267	— —		
15. NEPSY-II Total	0.574 **	0.337	0.484 **	0.406 *	NaN	0.174	0.107	−0.071	0.764 **	0.363 *	0.445 *	0.149	0.239	0.407	— —	
16. BVN Total	0.349	0.014	0.417 *	0.331	NaN	0.08	0.134	0.039	0.215	−0.005	−0.014	−0.132	−0.031	0.134	0.387 *	— —

*  *p* < 0.05, ** *p* < 0.001.

**Table 3:** Significant linear regression between hospitalization days and WPPSI-III subscales.

Dependent Variable	Predictors	B	SE	*p*	R^2^	95% Confidence Interval	
Hospital stay	Quotient (TQ) of the WPPSI-III scale	0.326	0.0652	0.015	0.893	0.344	1.55
Hospital stay	Verbal Intelligence Quotient (VIQ) of the WPPSI-III scale	0.223	0.0691	0.048	0.776	0.0125	1.75
Hospital stay	Overall score of the WPPSI-III scale	0.387	0.0915	0.024	0.856	0.229	1.62

**Table 4:** Paired samples *t*-test analyses.

	Sample	Mean	SD	*t*	df
Internalizing Problems	5-year-olds	5.38	5485	1552	0.125
	7-year-olds	7.69	5844		
Externalizing Problems	5-year-olds	7.27	5182	0.553	0.583
	7-year-olds	8.00	4787		
Total Problems	5-year-olds	12.52	7683	2.755	0.0007
	7-year-olds	20.21	12.451		

Note. Comparison between five- and seven-year-old children with respect to Internalizing, Externalizing and Total Problems of the CBCL 1.5–5 and CBCL 4–18.

## Data Availability

The dataset is not publicly available due the hospital privacy policy.
